# Purpura rhumatoïde de l’adulte dans le sud tunisien: une série de 14 cas

**DOI:** 10.11604/pamj.2019.34.107.17274

**Published:** 2019-10-23

**Authors:** Hanen Loukil, Mouna Snoussi, Faten Frikha, Moez Jallouli, Chifa Damak, Raida Ben Salah, Sameh Marzouk, Khaoula Kamoun, Mohamed Ben Hmida, Zouhir Bahloul

**Affiliations:** 1 Service de Médecine Interne, CHU Hédi Chaker, Sfax, Tunisie; 2 Service de Néphrologie, CHU Hédi CHaker, Sfax, Tunisie

**Keywords:** Purpura rhumatoïde, adulte, néphropathie, Henoch-Shonlein purpura, adult, nephropathy

## Abstract

Le purpura rhumatoïde (PR) est une vascularite des petits vaisseaux secondaire à des dépôts d'immunoglobulines de type IgA. À travers une étude rétrospective colligeant 14 cas de PR de l'adulte en se basant sur les critères de classification du PR de EULAR/ PRINTO/PRES durant une période de 18 ans (1996 à 2014) dans le service de médecine interne du CHU Hédi Chaker de Sfax dans le sud tunisien, les caractéristiques épidémiologiques, cliniques, thérapeutiques et évolutives du PR chez nos patients adultes seront précisées. L'âge moyen des patients était de 33 ans (extrêmes: 17-64 ans) avec une prédominance masculine (sex-ratio de 1.8). Le purpura vasculaire pétéchial était constant. Les manifestations articulaires (arthralgies et/ou arthrites) étaient notées chez 9 patients (64,2%). L'atteinte digestive était présente chez 13 patients (92.8%). L'atteinte rénale était notée chez 11 patients (78.5%) révélée par une protéinurie de rang néphrotique dans 7 cas, une hématurie microscopique dans 9 cas, une hypertension artérielle dans 4 cas et une insuffisance rénale dans un cas. Le type histologique le plus fréquent était la glomérulonéphrite proliférative endocapillaire diffuse (36.3%). La corticothérapie à forte dose était instaurée chez 7 patients ayant une atteinte rénale proliférative initiée par des bolus de solumédrol dans 4 cas et associée au cyclophosphamide dans un cas. Deux patients avec une atteinte digestive sévère ont reçu une corticothérapie. Après un suivi moyen de 18,5 mois (4-36 mois), l'évolution était favorable dans tous les cas sans rechutes et l'insuffisance rénale chronique était notée dans un seul cas.

## Abstract (en)

Henoch-Schonlein purpura (HSP) is a small-vessel vasculitis secondary to IgA deposits. We conducted a retrospective study of 14 cases of HSP in adults based on EULAR/PRINTO/PRES classification criteria for HSP over a period of 18 years (1996 to 2014) in Department of Internal Medicine at the Hedi Chaker University Hospital, Sfax, South Tunisia. The purpose of our study was to highlight the epidemiological, clinical, therapeutic and evolutionary features of HSP in our adult patients. The average age of patients was 33 years (ranging from 17-64 years) with male predominance (sex ratio of 1.8). Vascular purpura with petechiae was constant. Articular manifestations (arthralgia and/or arthritis) were reported in 9 patients (64.2%). Gastrointestinal involvement was reported in 13 patients (92.8%). Renal failure was found in 11 patients (78.5%) revealed by nephrotic proteinuria in 7 cases, microscopic haematuria in 9 cases, high blood pressure in 4 cases and renal impairment in 1 case. The most common histological type was diffuse proliferative endocapillary glomerulonephritis (36.3%). High dose corticotherapy was initiated in 7 patients with proliferative renal impairment, bolus of Solu-Medrol in 4 cases, associated with cyclophosphamide in one case. Two patients with severe gastrointestinal involvement received corticotherapy. After a mean follow-up period of 18.5 months (4-36 months), outcome was favorable in all cases without relapse and chronic renal failure was reported only in one case.

## Introduction

Le purpura rhumatoïde (PR) est une vascularite des vaisseaux de petit calibre, plus fréquente chez l'enfant que chez l'adulte, en rapport avec des dépôts de complexes immuns contenant des immunoglobulines A (IgA) [1]. Cette vascularite se caractérise par un purpura vasculaire, une atteinte articulaire, rénale et digestive. Le pronostic de la maladie dépend à court terme de la sévérité des manifestations digestives et à long terme des manifestations rénales [2]. A travers 14 observations de PR de l'adulte colligées dans le service de médecine interne du CHU Hédi Chaker de Sfax (Tunisie), nous préciserons les particularités épidémiologiques, cliniques, thérapeutiques et évolutives de cette vacsularite dans le sud tunisien.

## Méthodes

II s'agit d'une étude rétrospective transversale colligeant tous les cas de PR observés durant une période de 20 ans (janvier1996 à décembre 2016) dans le service de médecine interne du CHU Hédi Chaker de Sfax (Tunisie). Nos patients étaient tous âgés de 18 ans ou plus. Tous nos patients répondaient aux critères de classification du PR de EULAR/ PRINTO/PRES [3]. Le mode de début du PR était considéré comme aigu si les manifestations cliniques évoluaient depuis moins de 3 mois, subaigu si ce délai variait entre 3 et 6 mois et chronique s'il dépassait les 6 mois. L'atteinte rénale était retenue devant l'existence d'une protéinurie et/ou d'une hématurie microscopique et/ou une élévation du taux de la créatinine et/ou l'existence d'une hypertension artérielle récente. Nous avons utilisé la classification histologique des lésions glomérulaires du PR de l'adulte pour stadifier les lésions rénales [2]: Classe I: la glomérulonéphrite mésangiopathique ; classe II: la glomérulonéphrite proliférative segmentaire et focale; classe III: la glomérulonéphrite proliférative endo-capillaire diffuse; classe IV: la glomérulonéphrite proliférative endo- et extra capillaire; classe V: le rein fibreux, terminal. Les différentes explorations paracliniques étaient réalisées selon les signes fonctionnels du patient.

## Résultats

**Caractéristiques épidémiologiques:** notre série comprenait 9 hommes (64,3%) et 5 femmes (35.7%) avec un sex- ratio de 1.8 et une incidence annuelle de 0.77. L'âge moyen était de 33 ans avec des extrêmes allant de 18 à 64 ans. Sept patients étaient âgés de plus de 40 ans et un seul patient (7%) était âgé de plus de 60 ans. Aucun facteur déclenchant infectieux ou médicamenteux n'a été noté chez nos patients au moment de la poussé. Il n'y avait pas de pathologies auto-immunes associées au moment du diagnostic du PR ni au cours de l'évolution, par contre un carcinome bronchique était diagnostiqué 6 mois plus tard après la découverte de la vascularite chez un homme de la cinquantaine tabagique. Le mode de début du PR était aigu dans 10 cas (71.4%); subaigu dans 2 cas (14,2%) et chronique dans 2 cas (14,2%).

### Caractéristiques cliniques et paracliniques

**Manifestations cutanées:** le purpura vasculaire était constant. Il était surtout de type pétéchial (100%) compliqué de nécrose et d'ulcération dans 2 cas (14.2%) et associé à des lésions ecchymotiques dans un cas (7.14%). Le purpura touchait électivement les membres inférieurs de façon bilatérale et symétrique. Il était étendu dans 7 cas (50%) avec une atteinte du tronc et des membres supérieurs dans un cas respectivement. La biopsie cutanée était réalisée chez 12 patients objectivant une vascularite leucocytoclasique dans 11 cas parmi 12 (91.6%) et une infiltration lymphocytaire sans signes de vascularite dans un cas. L'immunofluorescence directe a pu être analysée chez deux patients révélant des dépôts vasculaires d'IgA.

**Manifestations articulaires:** les manifestations articulaires étaient retrouvées chez 9 patients (64.2%). Il s'agissait de polyarthralgies inflammatoires fugaces et migratrices des genoux et des chevilles. Seulement 5 patients avaient de véritables arthrites non destructrices au niveau des genoux (2cas), le coude (un cas), les chevilles (2 cas), les poignets (un cas) et les métacarpo-phalangiennes de façon unilatérale (un cas).

**Manifestations digestives:** elles étaient présentes chez 13 patients (92.8 %). Les douleurs abdominales d'allure colitique étaient constantes et conduisaient à une laparotomie blanche dans 2 cas. Cette douleur était associée par ordre de fréquence décroissante à des vomissements dans 5 cas (38,4%), des hémorragies digestives dans 4 cas (30.7%) à type de rectorragies et de méléna dans deux cas respectivement. Sept patients ont eu une échographie abdominale qui était pathologique dans 3 cas en objectivant une Infiltration œdémateuse de certaines anses intestinales avec un discret épanchement inter-anses chez 2 patients. Cinq patients ont eu un scanner abdominal ayant montré dans 4 cas un épaississement pariétal diffus des anses grêliques associé à un épanchement péritonéal dans 3 cas, un épaississement de la paroi gastrique dans un cas et une pancréatite aiguë dans un autre. L'entéro-IRM était réalisée une seule fois devant une atteinte grêlique diffuse, elle a révélé un épaississement pariétal circonférentiel et régulier, non sténosant, étendu sur toutes les anses du grêles, sans intervalle libre, épargnant la dernière anse iléale avec un œdème sous muqueux réalisant le signe de double halot en hyper signal T2. Huit fibroscopies digestives étaient pratiquées objectivant des lésions purpuriques gastriques chez 6 patients et des lésions nécrotico-hémorragiques duodénales chez 2 patients parmi 8 soit 25% des cas. Devant les rectorragies, deux colonoscopies étaient réalisées ayant montré des plaques congestives de la muqueuse colique chez un patient.

**Manifestations rénales:** l'atteinte rénale était notée chez 11 patients (78,5%).Les signes rénaux étaient: une protéinurie moyenne de 3g/24h (0.4-6g/24h) de rang néphrotique dans 7 cas (63.6%), une hématurie microscopique dans 9 cas (81.8%), une insuffisance rénale dans un seul cas (9%) et une hypertension artérielle dans 4cas (36.3%). La biopsie rénale était réalisée dans tous les cas montrant une glomérulonéphrite proliférative endo et extracapillaire (classe IV) dans 3cas (27.3%) une glomérulonéphrite proliférative endocapillaire diffuse (classe III) dans 4 cas(36.3%), une glomérulonéphrite segmentaire et focale (classe II) dans un cas (9%) et une glomérulonéphrite mésangiopathique (classe I) dans 3 cas (27.3%). L'étude en immunofluorescence avait montré des dépôts d'IgA dans le mésangium ou sur les parois des capillaires glomérulaires Tableau 1.

**Tableau 1:** Données démographiques, cliniques, histologiques, thérapeutiques et évolutives de l’atteinte rénale chez nos patients

Cas n°	Age	Sexe	Signes rénaux	Histologie	Traitement	Evolution
1	17	M	PU:2,16g/24h Hématurie HTA	Classe I	*Abstention*	*RC*
2	33	F	PU:4,26g/24h Hématurie	Classe III	*Corticothérapie*	*RC*
3	64	F	PU:1,35g/24h Hématurie HTA	Classe II	*Abstention*	*RP*
4	18	M	PU:0,42g/24h Hématurie	Classe IV	*Corticothérapie*	*RC*
5	49	M	PU:2,35g/24h Hématurie HTA	Classe III	*CorticothérapieCyclophosphamide*	*RC*
6	23	F	PU: 3g/24h	Classe I	*Abstention*	*RP*
7	50	M	PU:6,5g/24h Hématurie Insuffisance rénale	Classe IV	*Corticothérapie*	*RP*
8	57	F	PU:0,9g/24h HTA	Classe III	*Corticothérapie*	*RP*
9	53	M	PU:6g/24h Hématurie	Classe IV	*Corticothérapie*	*RC*
10	44	M	PU:3g/24h Hématurie	Classe I	*Corticothérapie*	*RC*
11	43	M	PU:2g/24h Hématurie	Classe III	*Corticothérapie*	*RC*

M: masculin F: Féminin PU: protéinurie, RC: rémission complète, RP: rémission partielle

**Manifestations biologiques:** la numération formule sanguine montrait une hyperleucocytose à PNN dans 13 cas (92.8%), une anémie inflammatoire et une thrombocytose dans 8 cas (57,1%). Un syndrome inflammatoire biologique était noté dans 9 cas (64.2 %). L''électrophorèse des protéines sériques montrait une hypogammaglobilinémie dans 3 cas (21.4 %) et une hypoalbuminémie en rapport avec une déperdition rénale dans 3 cas (21.4%). Une augmentation des IgA sériques était notée dans 7 cas (50 %) avec un taux moyen à 232 mg/l (extrêmes: 4,26 -600mg/l). Une augmentation du complément sérique était rencontrée dans 4 cas (28.5%) et des complexes immuns circulants étaient positifs dans un seul cas. Les anticorps anti cytoplasme des polynucléaires étaient recherchés chez tous les patients et se sont révélés négatifs.

**Traitement:** la corticothérapie était prescrite chez 9 patients (64.2%) et le cyclophosphamide chez un seul patient (7.14%). Deux patients ayant une atteinte digestive sévère avaient nécessité le recours à une corticothérapie à forte dose : un cas de pancréatite aiguë et un cas d'atteinte iléale diffuse résistante au traitement symptomatique. Concernant l'atteinte rénale, la corticothérapie était instaurée à forte dose chez 7 patients initiée par des bolus de solumédrol dans 4 cas associée au cyclophosphamide dans un cas.

**Evolution:** après un suivi moyen de 18 mois (1-36mois), l'évolution était favorable pour l'atteinte cutanée et articulaire avec une disparition des lésions cutanées dans un délai moyen de 15 jours (extrêmes : 7-32) et une résolution des arthralgies et des arthrites dans un délai moyen de 10 jours (extrêmes: 4-21). L'atteinte digestive était bénigne chez 11 patients parmi 13 avec une évolution spontanément favorable dans 3 cas et favorable sous traitement symptomatique (antispasmodique et /ou antiémétique) dans 8 cas et les corticoïdes ont été associés dans 2cas de présentation grave avec une évolution également favorable. L'évolution de la néphropathie s'est faite vers la rémission complète dans 7 cas (63.6%) (une fonction rénale normale et une négativation de la protéinurie) et la rémission partielle dans 4cas (36.6%) (une protéinurie séquellaire dans 3 cas et une insuffisance rénale chronique dans un cas ayant des lésions tubulointerstitielles à la biopsie rénale à côté de l'atteinte glomérulaire).

## Discussion

Le PR est vascularite à IgA rare chez l'adulte rapportée avec une incidence annuelle de l'ordre de 0.1 à 1.8 pour 100000 [2,4,5]. Le PR est de distribution ubiquitaire dans le monde avec une fréquence importante au Japon, en Asie du Sud-est, en Europe et en Australie [6]. En Afrique, le PR en particulier de l'adulte semble être beaucoup plus rare. En Tunisie son incidence est encore mal-connue. Notre étude monocentrique confirme le caractère rare du PR chez l'adulte avec une incidence annuelle de la maladie dans le sud tunisien estimée à 0.77. Aussi bien chez l'enfant que chez l'adulte, il existe une prédominance masculine de la maladie retrouvée également dans notre étude avec une sex-ratio de l'ordre de 1.8. Les facteurs déclenchants infectieux et médicamenteux ont été rapportés chez l'enfant. Par contre ce sont les néoplasies épithéliales muqueuses chez les tabagiques qui ont été rapportées comme facteur prédisposant chez l'adulte [6]. L'hypothèse pathogénique invoquée est une sécrétion d'antigènes tumoraux à l'origine d'une réponse anticorps spécifique anti-tumorale et ainsi la formation de complexes immuns, sur les parois capillaires, en particulier au niveau glomérulaire, réalisant de véritables néphropathies à IgA [7,8]. L'association d'un carcinome bronchique et d'un PR est notée dans un cas dans notre étude. Le PR de l'adulte diffère de celui de l'enfant non seulement par sa faible incidence mais aussi par la sévérité des manifestations cliniques [3]. L'atteinte cutanée était constante dans notre série et rarement nécrotique comparativement à d'autres séries asiatiques de PR de l'adulte ou l'atteinte cutanée est rapportée dans 73% des cas [9]. L'atteinte articulaire au cours du PR de l'adulte est fréquente, nous l'avons noté dans 64.3% des cas dans notre étude. Elle était moins fréquente dans d'autres études française et asiatique rapportée dans 50% et 44% des cas [9,10]. Dans les séries adultes ainsi que la nôtre, l'atteinte digestive au cours du PR est fréquente variant de 18 à 100% [10]. La douleur abdominale est le signe clinique le plus fréquent. Elle survient en moyenne chez 88% des patients ayant une atteinte digestive. Elle est associée par ordre de fréquence décroissante à une hémorragie digestive, une diarrhée, des nausées et des vomissements [6].

Les complications seraient plus fréquentes chez l'homme et restent rares: syndrome de malabsorption ou entéropathie exsudative [11,12]. Les explorations endoscopiques hautes montrent fréquemment des lésions purpuriques gastriques ou duodénales notées chez tous nos patients explorés parfois compliquées de nécrose et d'hémorragies dans 25% des cas. Notre étude confirme la supériorité du scanner abdominal par rapport à l'échographie abdominale dans la mise en évidence de l'épaississement inflammatoire de la paroi digestive, son étendu ainsi que l'atteinte d'organes pleins en l'occurrence le pancréas [6]. La prévalence de l'atteinte rénale est plus élevée au cours du PR de l'adulte 45 à 85%, [13,14] dans notre série elle était notée dans 78.5% au moment de la première poussée, l'hématurie microscopique étant le signe précoce notée dans 82% des cas souvent accompagnée d'une protéinurie parfois d'ordre néphrotique qui était constante chez nos patients. L'incidence de l'insuffisance rénale est élevée au cours du PR de l'adulte de l'ordre de 32% [3], elle était beaucoup plus rare chez nos patients soit 9% des cas. L'atteinte rénale semble être plus fréquente comme le confirme notre étude chez le sujet de sexe masculin. Au plan histologique, la lésion spécifique est une néphropathie glomérulaire avec des dépôts d'IgA mésangiaux longtemps identifiables dans les glomérules scléreux, permettant de porter un diagnostic rétrospectif [6]. Les lésions glomérulaires sont de sévérité variable, plusieurs classifications ont été établies, chez l'adulte la plus utilisée est celle proposée par Pillebout qui classe la néphropathie selon le degré de prolifération en 5 classes avec une grande corrélation anatomo-clinique [2]. Chez l'adulte, la lésion la plus fréquemment rencontrée est la glomérulonéphrite proliférative endocapillaire diffuse (classe III), la quatrième classe est rare dans moins de 10% des cas [6]. Les formes prolifératives diffuses étaient fréquentes chez nos patients (36.4%) et la classe IV était notée dans 27.3% des cas. Les lésions tubulo-interstitielles sont fréquemment associées aux lésions glomérulaires et sont fortement corrélées à la présence d'une insuffisance rénale [3]. Nous avons observé un seul cas d'insuffisance rénale chronique chez un patient ayant des lésions tubulo-interstitielles associées aux lésions glomérulaires. Des lésions vasculaires à type d'hypertrophie médio-intimale des petites artères, d'artériosclérose des artères interlobulaires et exceptionnellement d'une angéite nécrosante avec dépôts d'IgA des artérioles et petites artères peuvent se voir au cours du PR de l'adulte [6].

D'autres manifestations systémiques rares ont été rapportées au cours du PR, il s'agit des cas de myocardite, de péricardite ou de pancréatite aiguë [6]. Selon les études de la littérature, le PR de l'adulte se caractérise par la fréquence des rechutes (22%) et le passage à des formes chroniques (33%) [6]. Le pronostic à court terme chez l'adulte dépend de l'atteinte digestive et d'autres complications viscérales exceptionnelles de la vascularite: cardiaque, neurologique et pulmonaire [6]. Le pronostic à long terme est tributaire de l'atteinte rénale, en effet l'insuffisance rénale chronique est fréquente rapportée jusqu'à 68% [6]. Notre étude rétrospective trouvait une fréquence plus basse de l'ordre de 9%. Le Tableau 2 illustre le profil évolutif à long terme de l'atteinte rénale selon des séries de la littérature [10,15-20] . Parmi les facteurs de mauvais pronostic rénal ont été identifiés: une protéinurie au moment du diagnostic supérieure à 1 g/jour ou sa persistance au cours du temps [6,15], la présence d'une insuffisance rénale et d'une hypertension artérielle [3,15,21]. Ces différents éléments cliniques de mauvais pronostic ont été retrouvés chez le seul cas ayant présenté une insuffisance rénale chronique dans notre série. Le PR est une vascularite rare chez l'adulte et ses indications thérapeutiques sont mal codifiées. Les manifestations cutanées sont résolutives par le repos, les antalgiques et les anti-inflammatoires non stéroïdiens sont efficaces sur l'atteinte articulaire en l'absence de contre indications rénale ou digestive sévères [6]. Les principales indications des corticoïdes sont les manifestations digestives et rénales sévères. Chez l'adulte, aucune étude n'a pu à ce jour montrer l'efficacité des corticostéroïdes seuls pour prévenir l'évolution défavorable de l'atteinte rénale [15,22]. L'utilisation des immunosuppresseurs comme le cyclophosphamide et l'azathioprine au cours du PR est aussi mal précisée. Ces molécules semblent améliorer le pronostic des stades sévères de la glomérulonéphrite selon des séries pédiatriques [6]. Chez l'adulte, une étude multicentrique prospective randomisée incluant 54 patients adultes ayant un PR avec des atteintes viscérales graves n'a pas objectivé de bénéfice supplémentaire du cyclophosphamide seul par rapport aux corticoïdes seuls [20]. Les échanges plasmatiques et les immunoglobulines intraveineuses, seuls ou en association avec des stéroïdes et/ou des immunosuppresseurs, ont été proposés dans les formes graves [6]. Le rituximab a été utilisé avec succès au cours des formes sévères neurologiques et digestives résistantes aux corticoïdes et aux cyclophosphamide au cours du PR de l'enfant [23]. Chez l'adulte, cette molécule était efficace au cours d'une forme grave d'atteinte cutanée [24]. Dans notre étude la corticothérapie à forte dose a été utilisée dans les formes digestives graves (vascularite digestive étendue et pancréatite) ainsi que dans les glomérulonéphrites prolifératives (classe III et IV), le recours au cyclophosphamide a eu lieu dans un cas de glomérulonéphrite classe III avec une efficacité comparable aux corticoïdes seuls.

**Tableau 2:** Evolution rénale à long terme des patients adultes atteints de purpura rhumatoïde selon des séries de la littérature

Auteurs	Années	Nombre de cas	Patients avec atteinte rénale	Suivi (mois)	Nombre de rémission complète	Nombre d’IRC	Nombre d’IRCT
Lasseur *et al.*	1996	40	33	27 (3-156)	11	8	8
Coppo *et al.*	1995	95	95	12-180	31	15	15
Garcia-Porrua *et al.*	1980-2000	31	16	60 (16–245)	19	3	0
Rauta *et al.*	1980–1995	38	38	73,2 ± 4,3	NP	8	3
Coppo *et al.*	1995–2004	136	136	66 ± 36	NP	37	18
Uppal *et al*	1998–2003	20	18	33,6 ± 20,4	18	1	1
Pillebout *et al.*	2000-2009	54	51	60 (47–70)	NP	13	2
Notre série	1996-2016	14	11	18.5 (4-36)	6	1	0

## Conclusion

Le purpura rhumatoïde est une vascularite rare de l'adulte dans le sud tunisien. Les manifestations cliniques sont comparables à ceux de l'enfant avec une fréquence particulière dans notre étude comme dans la pluparts des séries de la littérature de l'atteinte rénale et digestive. Le PR de l'adulte dans notre région se caractérise par une évolution favorable des manifestations rénales et la faible fréquence de l'insuffisance rénale chronique et des rechutes à long terme. La corticothérapie reste le traitement de référence dans les formes graves avec une atteinte digestive étendue et des néphropathies glomérulaires prolifératives sans bénéfice supplémentaire du cyclophosphamide.

### Etat des connaissances actuelles sur le sujet

- Le purpura rhumatoïde est une vascularite systémique des petits vaisseaux en rapport avec des dépôts tissulaires de complexes immuns contenant des immunoglobulines A;
- Elle est rare et touche plus l'enfant que l'adulte surtout au Japon, en Asie du Sud-est;
- Le pronostic de la maladie dépend de l'atteinte digestive à court terme et de l'atteinte rénale à long terme.

### Contribution de notre étude à la connaissance

- Peu de séries dans la littérature qui se sont intéressées à l'étude du purpura rhumatoïde chez l'adulte. Notre étude est à notre connaissance la première dans la région nord africaine et en Tunisie qui s'est intéressée à ce sujet;
- Dans notre étude nous avons essayé de dégager les particularités cliniques du PR chez l'adulte jusque-là peu précisées par les séries de la littérature;
- Dans notre étude nous avons essayé de dégager le profil évolutif et pronostique du PR chez l'adulte dans notre région.

## Conflits des intérêts

Les auteurs ne déclarent aucun conflit d’intérêts.
